# Miscellaneous Cases

**Published:** 1842-10

**Authors:** Samuel Hogg

**Affiliations:** Late of Tennessee


Art. II.—*Miscellaneous Cases.* By Samuel Hogg, M. D., late of Tennessee.

[The following cases are taken from the Diary of Dr. Hogg, where they were recorded at various and distant periods, and probably without the most remote view to publication. They have nothing novel or particularly striking to recommend them, but they will be read with satisfaction by the physician engaged in practice. Y.]

J. M., aged 35 years, was taken on the 19th of July, 1814, with what he supposed to be bilious colic, having been subject to that complaint in former years. He had taken a cathartic when I visited him on the 22d, and was complaining of considerable nausea and burning heat in the stomach, fever, and frequent griping; his eyes and skin were yellow, his stools grey, and his urine high colored. I gave an emetic, which operated several times, but without any appearance of bile, and then moved his bowels, dislodging matter slightly bilious in its character. In the morning of the day on which I visited him, he had had a chill. He was ordered to take 5 grs. cal. every three hours until his bowels were acted upon, and on the return of fever next day to lose 8 ounces of blood.

23*d.* Calomel had not acted. Directed cal. & jal. aa. 15 grs. to be taken immediately, and to be repeated in six hours, if his bowels were not freely moved.

24*th.* Had a chill in the morning; powders had operated three times, bringing off dark green passages; fever high, and sense of burning in stomach persistent. Bled him to the extent of 10 ounces; the serum of the blood was in excess, and appeared largely mixed with bile, the crassamentum coagulating slowly. Ordered cal. in 8 gr. doses to be taken every six hours.

25*th.* The cal. had not operated. Chill repeated; burning heat in the epigastrium continues. He lost blood again, and took sulph. sod., which acted on his bowels, inducing dark, fetid evacuations. Ordered blister to the region of the liver.

26*th.* His chill had returned; pulse still active; blister very much inflamed; general yellowness increased. He lost half a pint of blood, which appeared to consist chiefly of bile and serum. Bowels have not been moved by the last calomel; nausea distressing. Left him 5 pills of cal. of 6 grs. each, to be taken once in six hours.

27*th.* Had taken four pills, which had produced some small passages of dark green matter. Directed him to take the remaining two.

28*th.* Saw him a few minutes after he had expired. An hour or two before he died, he vomited a considerable quantity of a dark colored matter mixed with blood.

*Case of Milk-sickness.*—On Tuesday night, 14th Dec. 1830, I was called to see Thomas Hunt, 30 years of age, who informed me that he lived on Goose Creek, in Sumner County, and that some of his family had recently been affected with what was called milk-sickness, in consequence, it was supposed, of their having used the milk of cows laboring under the trembles. He left home for Nashville on Thursday, the 9th, and was seized, after much fatigue, with symptoms of the same disease. The symptoms were, pain in the head, chilliness, nausea, irritability of stomach and vomiting. When I saw him five days after he was attacked, his bowels had not been evacuated; sense of burning in the stomach and bowels insupportable; vomiting on taking any thing into the stomach, the matter ejected being of a greenish color, and of a peculiar odor, which he said was an invariable attendant of milk-sickness. His tongue moist, clean, smooth and of natural color; general surface dry, cool, and rather livid, features shrunk, and indicating extreme distress; pulse 110 in the minute, and laboring.

*Treatment.* Venesection was attempted, but the sinking of his system compelled me soon to close the orifice. Ol. ricina. with 40 gt. tinct. opi. was administered, but not long retained. Fomentations of the abdomen and extremities were adopted, and pills of cal., rhu. & al. were directed in doses to move his bowels. Next morning he had taken four pills, but without the desired effect. Symptoms more alarming; patient becoming comatose, but when roused gives correct answers; great thirst; says his bowels are burning up; passes no urine. Enemata being used, he passed some very hard, dark fecal matter and bloody sanies. Cal. 30 grs. & tinct. opi. 40 grs. were administered and retained. Sinapisms were applied to the stomach and extremities. At 12 o'clock, M., 10 drops croton oil were given, and at 2 P. M. the dose was repeated without effect. Difficulty of swallowing is becoming great, pulse sinking, great chilliness. 4 o'clock. Patient is evidently sinking. Takes strong tea of rad. serpent. with paregoric as long as able to swallow. Expired about 12 at night.

*Cases in Midwifery.* Mrs. M., a lady of delicate habit, was taken with the usual signs of approaching labor on a Thursday morning; the midwife waited on her till Friday evening, and labor then not advancing, I was called in. I found her with considerably increased arterial action, the os tincæ but little dilated, and the child apparently large. She lost at once a pint of blood, and took 40 drops of laudanum. Her pains came on regularly, which had not previously been the case, but the os tincæ continued rigid, and the child advanced very slowly. In about five hours she was again bled to the extent of three gills, and the opiate was repeated.

The os tincæ in about three hours now began to dilate, and the membranes being found on examination to be protruded, were immediately ruptured and the waters passed off. I now found the head, which was very large, just entering the passage, and rather confined between the sacrum and pubis. Her pains began to decline, and as she was much exhausted by the labor and the officiousness of the midwife, I concluded to allow her an opportunity of taking some rest, and accordingly ordered her to be put to bed and not disturbed till necessity should compel her to rise. She slept an hour, and on waking the pains returned with great force, and in about an hour she was safely delivered of a very large boy. She had borne three children previously, with two of whom she was obliged to have assistance. The blood drawn showed on cooling considerable buffiness.

A negress of stout frame and good health was seized with labor pains on a Monday morning, having given birth to one child before. Her pains were irregular and not very strong, but in the evening, the midwife being anxious to deliver, ruptured the membranes with her finger, whereupon the pains ceased, and I was called in. I found her resting quietly, though much fatigued, and complaining of soreness, the result of excessive handling; the os uteri was rigidly contracted, and the head barely in reach of the finger. Her pulse being active, I took a pint of blood, gave her an opiate, and directed that she should be allowed to rest. She slept about three hours, and on awaking she had some pain, but as they were inefficient, I again abstracted blood from her arm. I left her at 10 o'clock at night, and charged the attendants not to interrupt her if she were inclined to sleep. In the morning I was summoned in haste by the midwlfe, who insisted on my opening the head of the fœtus, as she concluded it to be dead, and thought the woman could not be delivered without it. On examination I found that the head had advanced a little, but the os uteri was still rigid, and the strength of the patient being unimpaired, I drew blood a third time. In half an hour she had a call to the close stool, where she was seized with a severe pain, and on examining her soon afterwards, the head was found nearly cleared, and the next pain expelled the child alive. I then waited a few minutes, when the placenta, after a slight pain, was safely expelled, and the woman afterwards did well.

Mrs. J. S. A., a woman of robust constitution and mother of six children, was taken on a Friday with the usual signs of approaching labor. When the midwife was sent for about 4 o'clock in the morning, the labor was well advanced, and about 11 o'clock, A. M., she was delivered of a boy. Immediately after the birth of the child the midwife proceeded to clear her, although she appeared still to be unusually large; but the placenta being firmly fixed the cord was ruptured in the effort to force it away. In the course of the next hour the midwife made frequent attempts to get it away, but failing, she became alarmed and sent for me. When I arrived I found the woman quite easy, but much alarmed with the idea of a false conception, which I soon dissipated by an examination which disclosed the presence of another child. As it was high in the pelvis I abstracted a little blood, and giving a few drops of laudanum, exhorted her to patience. In an hour, having a call to the urinal, she distinctly perceived the motion of the child. Finding that she was in no danger, the old woman having fortunately failed to dislodge any portion of the placenta, I suffered her to rest until sunset, some six hours after the birth of the first child, when, her pulse being active and the friends pressing that something should be done to expedite delivery, I took a little more blood. Next morning about 10 o'clock her pains began to return, but were weak. I gave her 40 drops of laudanum, with a tea-spoonful of tinct. camphor, which had the effect of increasing the pains, and at 1 o'clock the head was found far advanced. The child, a second boy, was born in half an hour afterwards, but was saved with difficulty in consequence of the cord having been wound twice around its neck so as nearly to stop the circulation. In a few minutes a single placenta was expelled without hemorrhage, notwithstanding that the cord had been ruptured and the umbilical vessels open for twenty-seven hours. The woman did well.

